# Antidepressant effect and neural mechanism of *Acer tegmentosum* in repeated stress–induced ovariectomized female rats

**DOI:** 10.1080/19768354.2020.1808063

**Published:** 2020-08-18

**Authors:** Hyun-Jung Park, Hyun Soo Shim, SongYi Park, Insop Shim

**Affiliations:** a Department of Physiology, College of Medicine, Kyung Hee University, Dongdaemun-gu, Republic of Korea; b Department of Food Science & Biotechnology, College of Science and Engineering, Kyonggi University, Suwon-si, Republic of Korea

**Keywords:** *Acer tegmentosum*, repeated stress, cytokine, ovariectomy, tyrosine hydroxylase (TH)

## Abstract

*Acer tegmentosum* (ATM) has antioxidant and anti-adipogenic activity. However, few studies have investigated the pharmacological activity or mechanism of ATM as an antidepressant agent. We assessed the antidepressant effect of ATM in modulating menopausal depressive symptoms and its mechanisms in ovariectomized (OVX) and repeatedly stressed (RS) female rats. The female rats were randomly divided into four groups: (1) naïve normal (normal) group, (2) OVX + repeated stress + saline-treated (control) group, (3) OVX + repeated stress + ATM (100 mg•kg^−1^)-treated (ATM100) group and (4) OVX + repeated stress + ATM (400 mg•kg^−1^)-treated (ATM400) group. We performed a battery of tests, such as the forced swimming test (FST), the sucrose intake test, and social exploration. After behavior testing, serum corticosterone levels were examined, followed by immunohistochemical determination of c-Fos, tyrosine hydroxylase (TH), and interleukin-1 beta (IL-1β) expression in the brain. ATM administration was associated with significantly decreased immobility time in the FST. Also, the control group tended to have decreased sucrose intake and social exploration compared with the normal group. However, ATM treatment was associated with markedly increased sucrose intake and active social exploration. In the paraventricular nucleus, c-Fos and IL-1β expression were significantly decreased in the ATM400 group compared with the control group. Compared with the control group, high-dose ATM administration was also associated with markedly decreased expression of TH-immunoreactive neurons in the locus coeruleus. The study findings demonstrated that ATM treatment effectively decreased behavioral and pathophysiological depression-like responses.

## Introduction

Emerging evidence indicates that proinflammatory cytokines, including interleukin (IL)-1, IL-6, and tumor necrosis factor-alpha (TNF-α), contribute to the pathophysiology of depression (Szkaradkiewicz et al. 2009; Grivennikov and Karin 2011; Felger and Lotrich 2013). Hypersecretion of proinflammatory cytokines activates immune responses and leads to continuous increases in cortisol secretion in the context of clinical depression (Jeon and Kim 2018). Recent studies have demonstrated that proinflammatory cytokines are involved in monoamine pathways (Park et al. 2015; Lee et al. 2016). Some proinflammatory cytokines play similar roles in depression-related pathophysiological mechanisms (Bauer and Teixeira 2018; Jeon and Kim 2018; Lezheiko et al. 2018). We previously demonstrated increased IL-1β activation in the paraventricular nuclei of repeated restraint–stressed rats (Park et al. 2014).

Menopause is associated with a rapid decline in estrogen secretion. On average, women spend more than a third of their lifetime in the postmenopausal period, and estrogen deficiency is a major cause of depression among women. Many recent studies have focused on the hypothalamic-pituitary-ovarian axis, wherein the action of women’s sex hormones has been believed to be restricted in the context of depression (Vegeto et al. 2002). Accumulating evidence suggests an association between major depressive episodes and inflammatory response activation (Vegeto et al. 2002).

*Acer tegmentosum* Maxim. (Aceraceae) (ATM) is a type of deciduous tree that grows in Korea, Russia, and the northern areas of China. ATM has been shown to have antiangiogenetic, anti-atopic, and radical-scavenging effects. Yu et al. (2010) demonstrated the ethnopharmacological capacity of ATM against hepatic inflammatory activity both in vitro and in vivo. *Acer* species have been used in ethnomedical practice for their antimicrobial, antidiabetic, anti-inflammatory, and hepatoprotective effects as well as their ability to improve osteoblast differentiation (Ho et al. 2012). However, few studies have investigated the pharmacological activity or mechanism of ATM against depression induced by neuroinflammation.

We aimed to investigate the antidepressant effects of ATM in modulating menopausal depressive symptoms and its mechanisms in ovariectomized (OVX) and repeatedly stressed female rats. We modeled depression-like behavior in rats using social exploration, sucrose intake, and the forced swimming test (FST). We assessed serum corticosterone (CORT) levels and brain expression of c-Fos, IL-1β, and tyrosine hydroxylase (TH).

## Methods

### Subjects and surgery

Three-month-old female Sprague–Dawley rats (Orient, Inc. Korea) rats were housed under a controlled temperature (22–24°C, 30%–70% relative humidity) with a 12 h light/dark cycle. The lights were on from 08:00–20:00. Food and water were made available ad libitum. The rats were allowed at least 1 week to adapt to their environment before the experiments. All experiments were approved by the Kyung Hee University institutional animal care and use committee (KHUASP (SE)-13-014). The rats were randomly divided into four groups: (1) naïve normal (normal) group, (2) OVX + repeated stress + saline-treated (control) group, (3) OVX + repeated stress + ATM (100 mg•kg^−1^)-treated (ATM100) group and (4) OVX + repeated stress + ATM (400 mg•kg^−1^)-treated (ATM400) group. Bilateral ovariectomy was performed under aseptic conditions and general anesthesia with pentobarbital sodium (50 mg/kg, i.p.). After 7 days of postoperative recovery, the OVX rats were stressed daily. ATM was orally administered immediately before each stress session. Stress was produced by forcing the animals into an immobilization device (a disposable rodent restraint cone, Yusung, Korea) for 2 h (10:00–12:00 am) each day for 14 days.

### Preparation of ATM extracts

The dried ATM samples (200 g, Jungdo, Seoul, Korea) were immersed in a 10-fold volume of dH2O, boiled at 80°C for 1 h, then the water extract was collected. The process was repeated once, and the extracts were combined and concentrated with a rotary evaporator and vacuum-dried to yield 9.9% (w/w) of the extract.

### Behavioral testing

#### FST

The FST was originally described by Porsolt et al. (1977) (Yirmiya et al. 1996; Bluthe et al. 2002; Bekris et al. 2005) and is now the most widely used test for assessing antidepressant activity in laboratory animal models (Dalla et al. 2005). The development of immobility when the rodents are placed in an inescapable cylinder of water reflects the cessation of persistent escape-directed behavior (Maier and Watkins 1998). The apparatus consisted of a transparent Plexiglas cylinder (50 cm high × 20 cm wide) filled to a 30 cm depth with water at room temperature. During the pre-test, the rats were placed in the cylinder for 15 min, 24 h before the 5-min swimming test. During the 5-min swimming test, the following behavioral responses were recorded by a trained observer: climbing behavior, defined as upward-directed movements of the forepaws along the side of the swim chamber; and swimming behavior, defined as movement throughout the swim chamber, including crossing into another quadrant. A rat was considered immobile when it made no further attempts to escape but made movements necessary to keep its head above the water. Increases in active responses, such as climbing or swimming, and reductions in immobility, were considered to reflect antidepressant-like activity.

### Social exploration

Behavioral observations were carried out during the dark phase of the light/dark cycle, under red-light illumination. This included assessments of depressive behavior among the rats induced by repeated stress. Rats were introduced into the home cage of the test animal for 5 min (Yirmiya et al. 1996; Bluthe et al. 2002). One day before the experiment, baseline social exploration was assessed. The total time spent by the experimental rat in social exploration during the 5-min session was recorded by a skilled observer blinded to the experimental conditions. The observer recorded the total time that the experimental rat was in contact with a juvenile rat elsewhere in the experimental field; only contact that was directly initiated by the experimental rat was defined as ‘social exploration’ (e.g. anogenital and body sniffing, grooming of the juvenile, licking). Leaning or incidental side-to-side touching was not counted.

### Sucrose intake

This experiment was designed to assess the preference for a palatable solution using a two-bottle paradigm in which rats could freely choose between a bottle of water and a bottle containing a mild 1% (w/v) sucrose solution. Blunted sucrose intake in this test has been proposed to mirror an impaired sensitivity to reward and to model anhedonia, a core symptom of major depressive disorder (Yirmiya et al. 1996; Dalla et al. 2005). During the week preceding the initiation of the experiments, rats were trained to consume the mild 1% (w/v) water sucrose solution to ensure stabilized baseline sucrose consumption. Training consisted of seven 12-h tests (beginning just as the lights were switched off), and the animals could freely select between two preweighed bottles: one with sucrose solution and one with tap water. Rats were neither food-deprived nor water-deprived before or during the test. The positions of water and sucrose bottles in the cage were alternated daily to prevent the rats from developing a place preference. Fluid consumption (g) was measured by weighing the bottles before and after each test session.

### Enzyme-linked immunosorbent assay (ELISA) for CORT detection

After all behavioral tests, animals were deeply anesthetized with sodium pentobarbital (80 mg/kg, i.p.). Serum samples were then drawn from the heart and centrifuged (3000 g, 15 min) at 4°C. All serum samples were frozen at −70°C until used for measuring biochemical variables.

We collected blood samples from the rats immediately after the behavior testing. The total concentration of CORT was measured using an ELISA kit (ELISA Development System, R&D Systems, Inc., Minneapolis, MN, USA). Cardiac blood was collected just before sacrificing the rats. The blood was centrifuged for 15 min at 1000 × g within 30 min of collection. The samples were immediately assayed or stored at ≤−60°C. All of the reagents, working standards, and samples were prepared in advance. The excess microplate strips were removed from the plate frame and returned to the foil pouch containing the desiccant pack, and then the pouch was sealed. All of the samples or standards (100 μL) were added to the appropriately labeled wells, and 50 μl of conjugated serum was placed into all of the wells except for the nonspecific binding wells and the total count wells. CORT (50 μL) was added to all of the wells. All of the wells were incubated for 2 h at room temperature on a horizontal orbital microplate shaker (0.12″ orbit) set at 500 ± 50 rpm. Each well was washed three times with wash buffer. After the last washing, any remaining wash buffer was removed by aspirating or decanting. Then, 5 μL of CORT conjugate and 200 μL of *p*-nitrophenyl phosphate-substrate were added to all of the wells. The wells were incubated for 1 h at room temperature (without shaking). Next, 50 μL of stop solution was added to each well. The optical density of each well was immediately determined using a microplate reader. Absorbance was read at 450 and 550 nm, and the sample values were calculated from a standard curve.

### c-Fos and (IL-1β) and TH immunohistochemistry

Immediately after the behavioral tests were completed, the animals were anesthetized with sodium pentobarbital (100 mg/kg, i.p.). The rats were perfused transcardially with 100 mL of saline followed by 500 mL of a 4% solution of formaldehyde prepared in phosphate-buffered saline (PBS). The brains were then removed, post-fixed in the same fixative for 2–3 h at 4°C, and then placed overnight at 4°C in PBS containing 20% sucrose. The following day, the brain was cut into coronal sections and further sliced into 30-µm thick sections. Sections were processed for c-Fos immunoreactivity using rabbit c-Fos polyclonal antibody (c-Fos, concentration 1:2000; Santa Cruz Biotechnology, Santa Cruz, CA, USA), IL-1β immunoreactivity using rabbit-IL-1β polyclonal antibody (IL-1β, concentration 1:100; R&D Systems, Inc., Minneapolis, MN, USA), or TH immunoreactivity using mouse-TH polyclonal antibody (TH, concentration 1:2000; Santa Cruz Biotechnology, Santa Cruz, CA, USA). The sections were then processed by a conventional avidin–biotin-peroxidase method (Vector Laboratories, Burlingame, CA, USA). The tissue was developed using diaminobenzidine (Sigma, St. Louis, MO, USA) as the chromogen. The sections were mounted on gelatin-coated slides, air-dried, and placed between a coverslip and slide for microscopic analysis. A micro rectangular grid (200 × 200 μm) was placed on the paraventricular nucleus (PVN) area, according to the atlas of Paxinos and Watson (Paxinos et al. 1985), for measuring the cells under a light microscope (×100 magnification).

### Statistical analysis

Statistical comparisons were performed for the behavioral and histochemical studies using the one-way ANOVA and least significant difference (LSD) post hoc test**.** All of the results are presented as means ± standard error of the mean We used SPSS Statistics for Windows, version 15.0 (SPSS Inc., Chicago, IL, USA) for statistical analyses. The significance level was set at *P *< 0.05.

## Results

### FST observations

**Figure 1.**
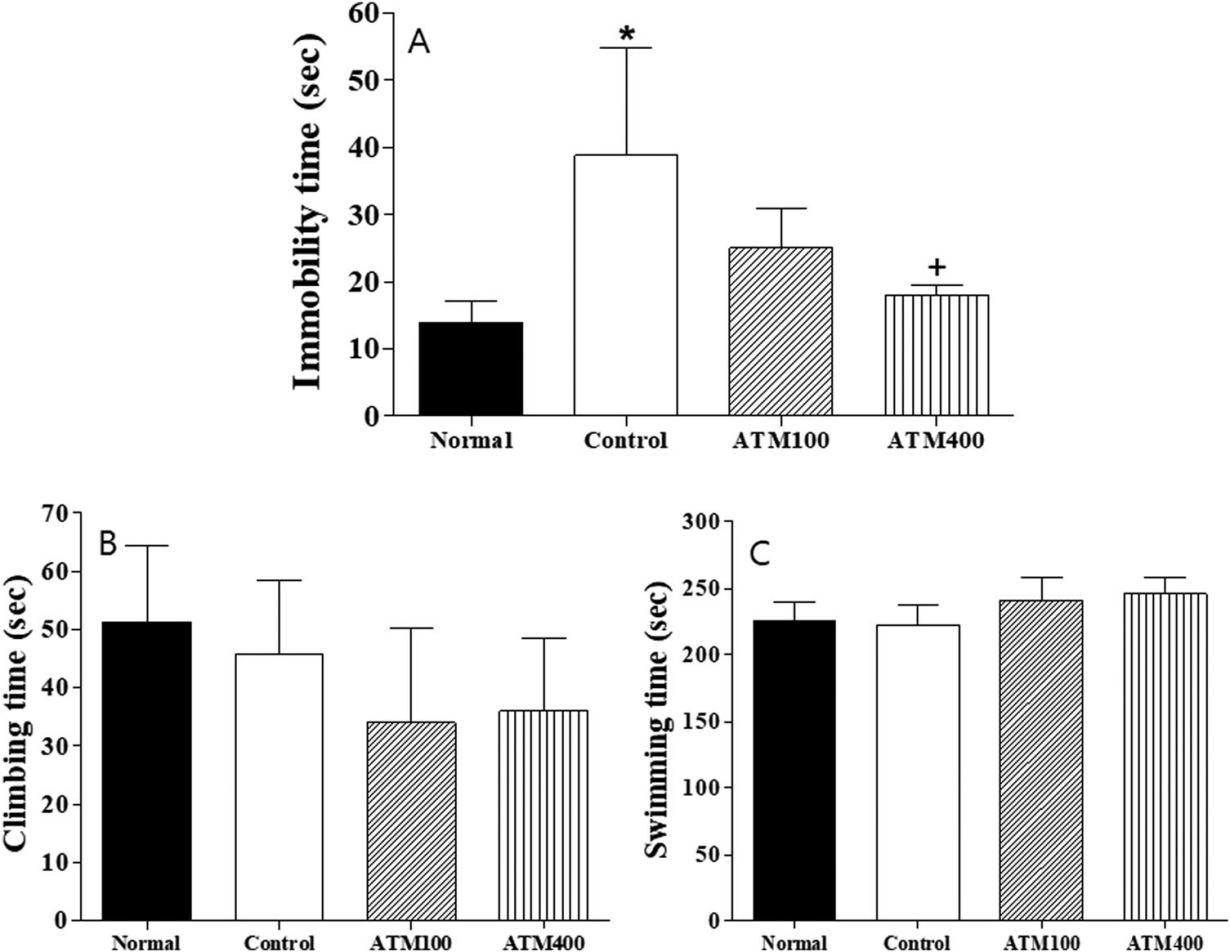
The effects of *Acer tegmentosum* (ATM) extract on the forced swimming test (FST) in the rats. Data represent means ± standard error of the mean (SEM) of the duration of (A) immobility, (B) climbing, and (C) swimming during the 5-min test session. Note: The results of FST were analyzed by performing separate one-way ANOVA of mean counts among the groups. Each value represents the mean ± SEM. *P < 0.05 compared with normal and ^+^*P *< 0.05 compared with control.

**Figure 2.**
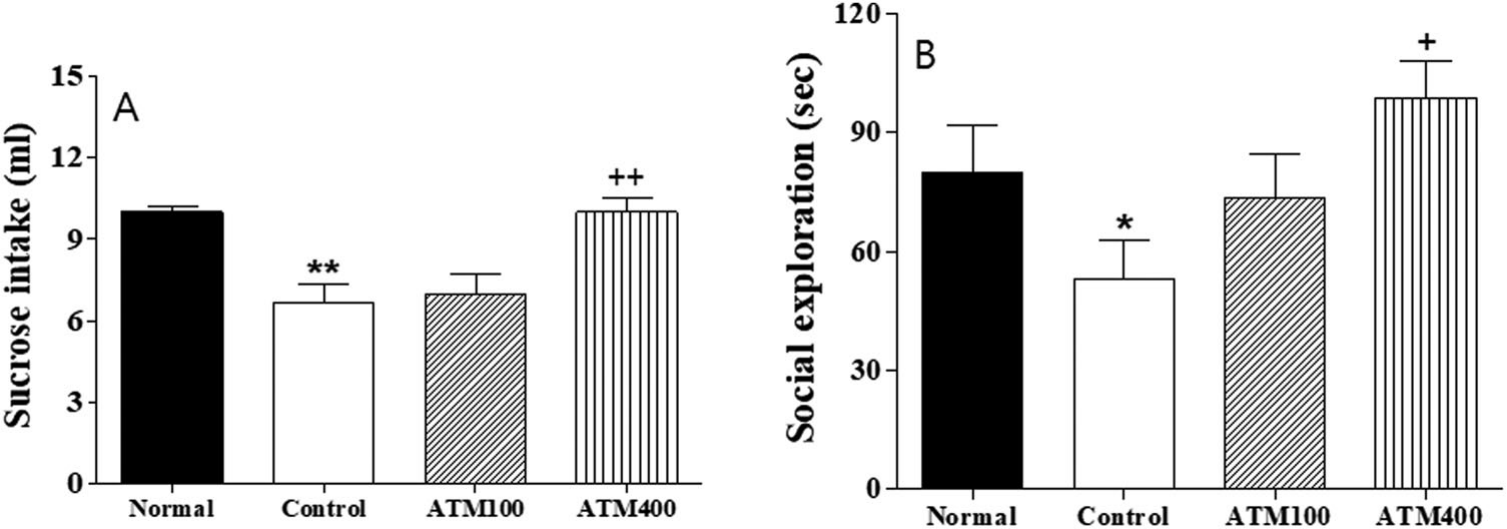
The mean ± standard error of the mean (SEM) values of (A) sucrose intake and (B) social exploration in the rats. Note: The results of the sucrose intake and social exploration experiments were analyzed by performing separate one-way ANOVA of mean counts among the groups. Each value represents the mean ± SEM. *P < 0.05, **P < 0.01 compared with normal and ^+^*P *< 0.05, ^++^*P *< 0.01 compared with control.

We used the FST to evaluate the ability of rats to cope with a stressful and inescapable situation (learned helplessness). As shown in Figure 1A-C, the animals displaying increased immobilization periods were considered to be exhibiting increased helplessness and depression-like behavior. There was statistical difference among the groups (F_3, 23 _= 7.8, *P *< 0.05; Figure 2A). The mean immobility time was significantly decreased in the ATM400 group compared with the control group (*P* < 0.05).

### Sucrose intake

**Figure 3.**
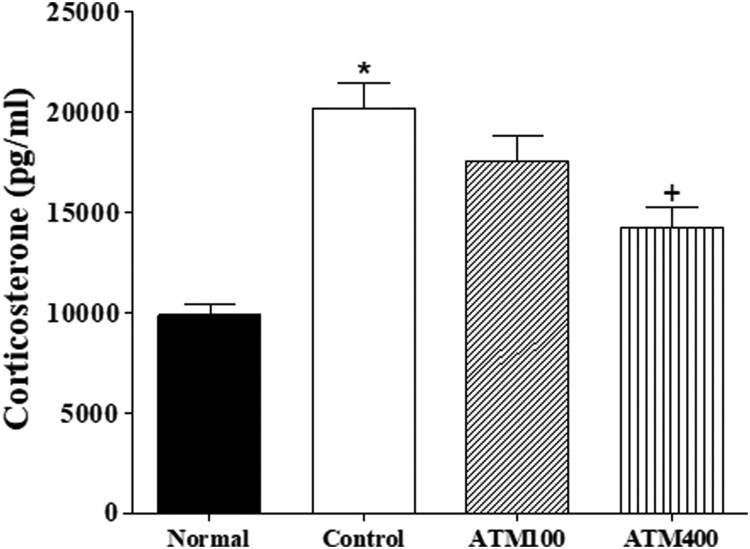
The mean ± standard error of the mean (SEM) values of corticosterone in the serum. Note: The ELISA results were analyzed by performing separate one-way ANOVA of mean counts among the groups. Each value represents the mean ± SEM. *P < 0.05 compared with normal and ^+^*P *< 0.05 compared with control

In terms of the anhedonic response, sucrose intake significantly differed among the groups (F_3, 2 _= 6.7, *P *< 0.01; Figure 3A). The LSD test results indicated that the mean sucrose intake was significantly lower in the control group compared with the normal group (*P *< 0.01). However, the mean sucrose intake among rats in the ATM400 group was significantly higher than that among rats in the control group (*P *< 0.01).

### Social exploration

The social exploration test results also significantly varied among the groups (F_3, 23 _= 9.9, *P *< 0.05; Figure 3B). The LSD test results indicate that the social exploration behavior was decreased in the control group compared with the normal group (*P *< 0.05). Moreover, the social exploration behavior of the ATM400 group was significantly increased compared with that of the control group (*P *< 0.05).

### Serum CORT levels

**Figure 4.**
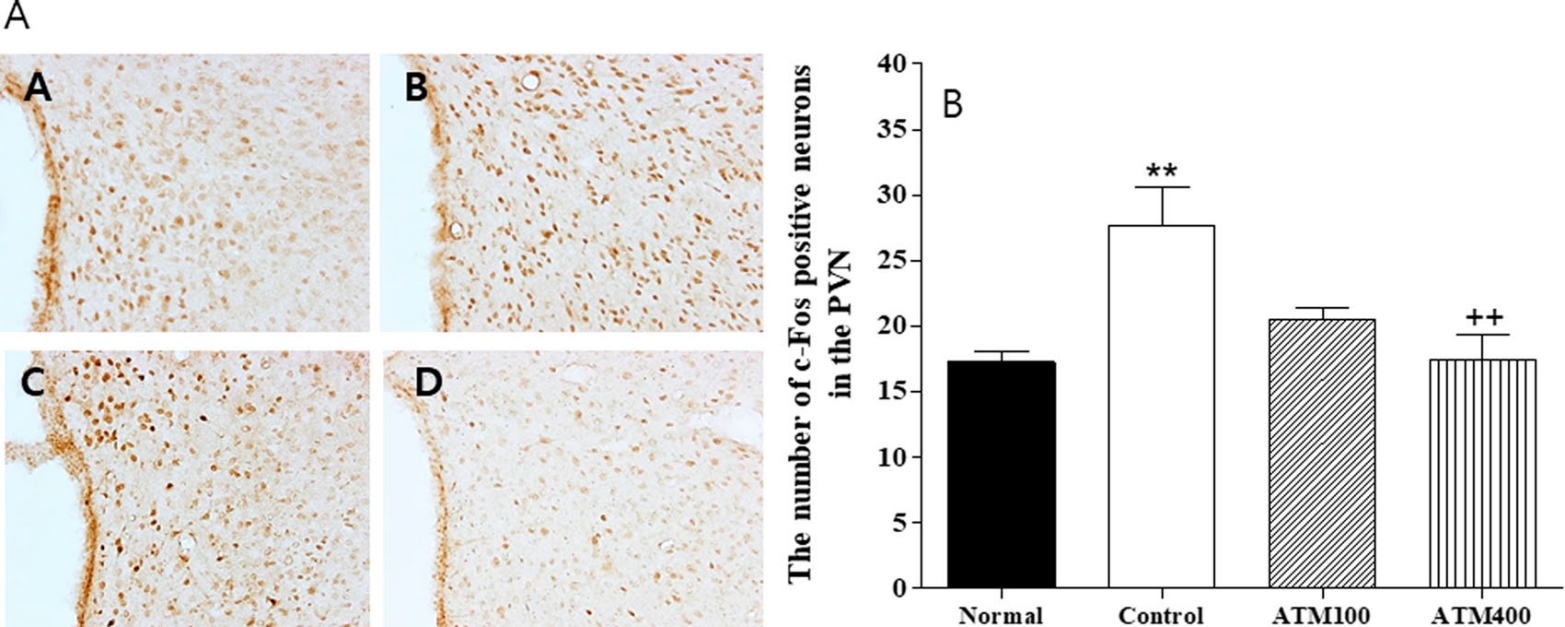
**A:** The mean ± standard error of the mean (SEM) values of quantities of c-Fos–immunostained nuclei in the paraventricular nucleus (PVN) of the experimental groups **B:** Photographs showing the distribution of c-Fos immunoreactive cells in the PVN of the (A) normal group, (B) control group, (C) ATM100 group, and (D) ATM400 group. Coronal sections were 30 μm thick, and the scale bar represents 50 μm (100 × 100). Note: The immunohistochemistry results were analyzed by performing separate one-way ANOVA of mean counts among the groups. Each value represents the mean ± SEM. **P < 0.01 compared with normal and ^++^*P *< 0.01 compared with control.

Serum CORT levels significantly varied among the groups (F_3, 23_ = 8.4, *P *< 0.05; Figure 4A). The LSD test results indicate a significantly lower mean CORT level in the control group compared with the normal group (*P* < 0.05). The mean CORT level of the ATM400 group was significantly lower than that of the control group (*P* < 0.05).

### Immunohistochemistry

#### c-Fos

The paraventricular c-Fos expression findings are shown in Figure 4A and B. The mean c-Fos neuron count in the PVN area was 17.3 ± 0.8 in the normal group, 27.7 ± 2.9 in the control group, 20.5 ± 0.9 in the ATM100 group, and 17.5 ± 1.8 in the ATM400 group (F_3, 23 _= 6.7, *P* < 0.01). The mean c-Fos neuron count of the ATM400 group 63.2% that of the control group (*P* < 0.01), meaning that the administration of 400 mg/kg ATM was associated with a downregulation of c-Fos positive neurons in the PVN.

#### TH

**Figure 5.**
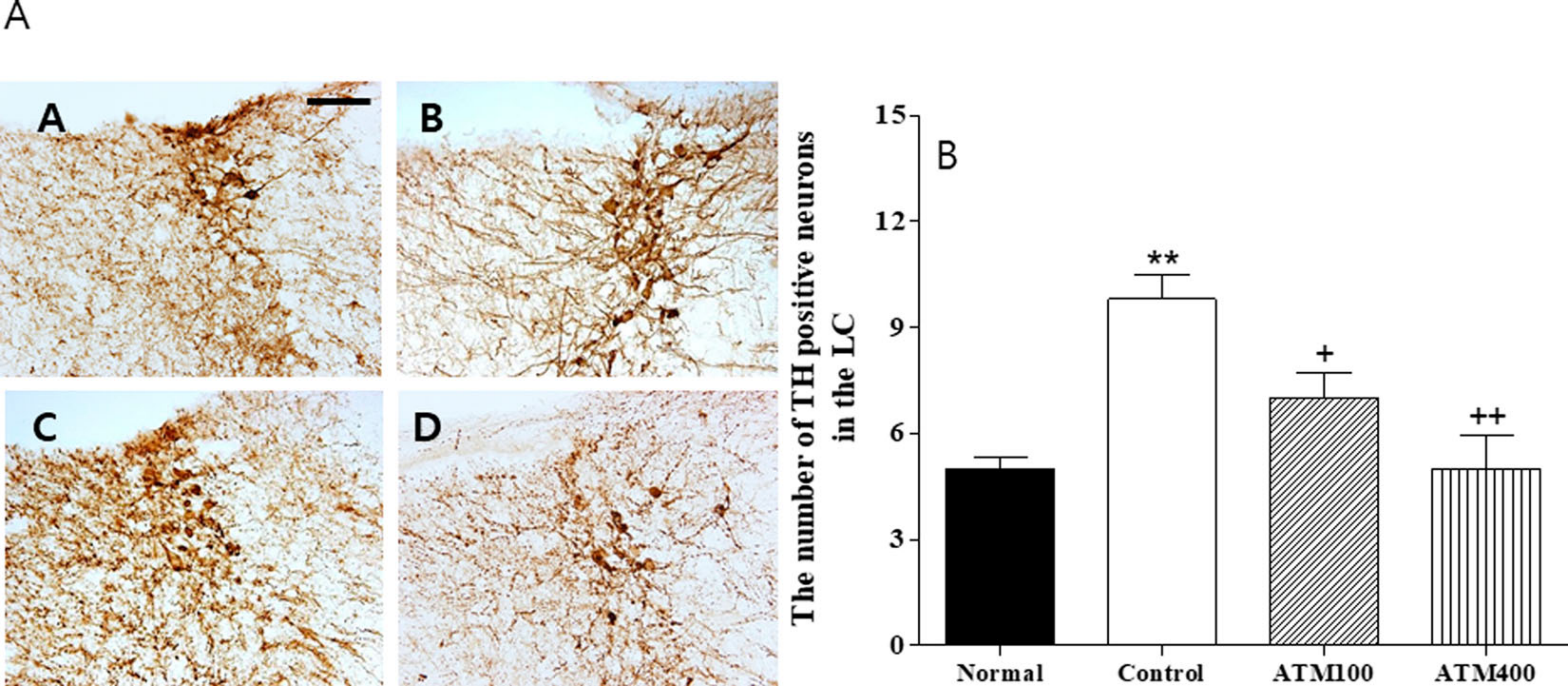
**A:** The mean ± standard error of the mean (SEM) values of quantities of tyrosine hydroxylase (TH)–immunostained nuclei in the locus coeruleus (LC) of the experimental groups **B:** Photographs showing the distribution of c-Fos–immunoreactive cells in the LC of the (A) normal group, (B) control group, (C) ATM100 group, and (D) ATM400 group. Coronal sections were 30 μm thick, and the scale bar represents 50 μm (100 × 100). Note: The immunohistochemistry results were analyzed by performing separate one-way ANOVA of mean counts among the groups. Each value represents the mean ± SEM. **P < 0.01 compared with normal and ^+^*P *< 0.05, ^++^*P *< 0.01 compared with control.

The evaluation of the TH-immunoreactive cells per section of the paraventricular area are shown in Figure 5A and B. The mean TH-positive neuron count in the locus coeruleus (LC) area was 5.0 ± 0.3 in the normal group, 9.8 ± 0.7 in the control group, 7.0 ± 0.7 in the ATM100 group, and 5.0 ± 0.9 in the ATM400 group (F_3, 23 _= 10.3, *P* < 0.001). In the normal group, the mean TH-positive neuron count was 200.0% that of the controls group (*P* < 0.01). The LSD post hoc test revealed that the mean TH-positive neuron count in the LC was significantly lower in the ATM-treated groups than in the control group (ATM100: *P* < 0.05, ATM400: *P* < 0.01). Therefore, ATM administration was associated with suppression of TH-positive neurons in the LC.

#### IL-1β

**Figure 6.**
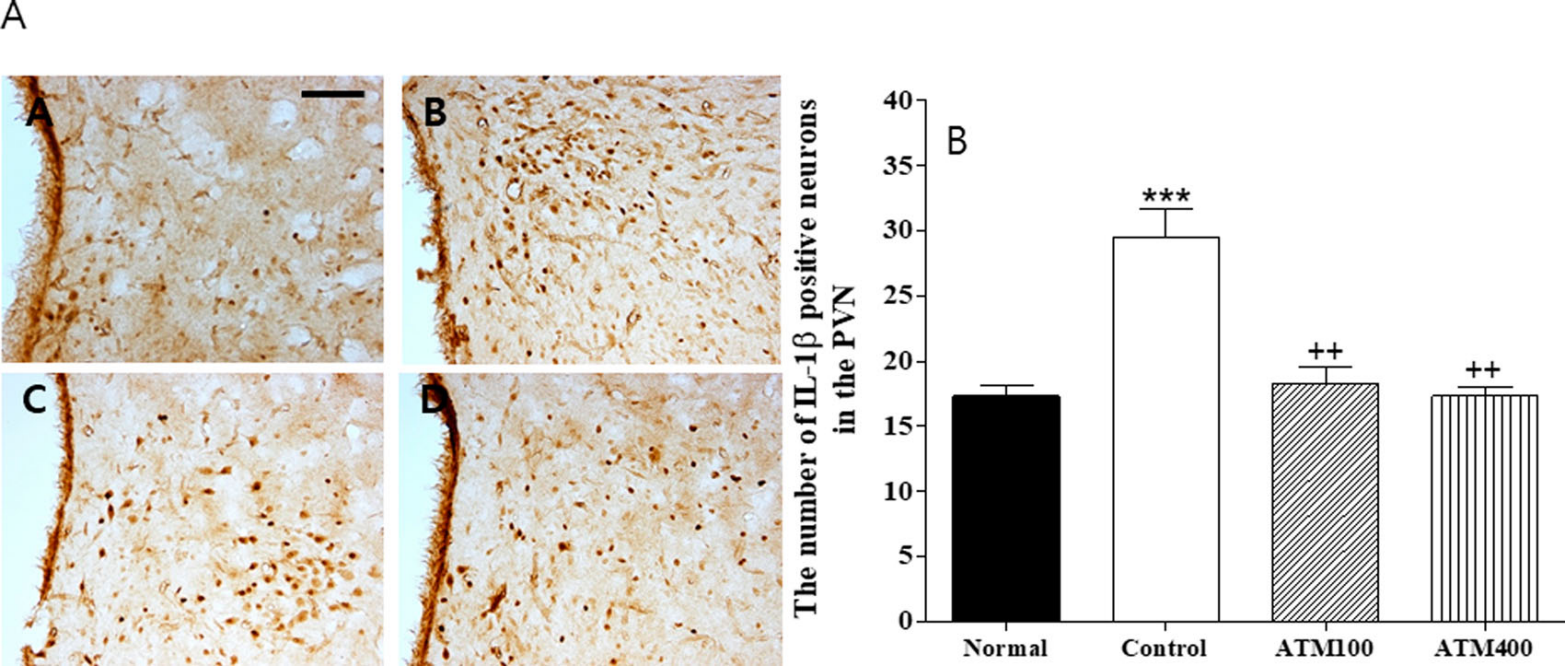
**A:** The mean ± standard error of the mean (SEM) values of quantities of IL-1β–immunostained nuclei in the paraventriular nucleus (PVN) of the experimental groups **B:** Photographs showing the distribution of IL-1β–immunoreactive cells in the PVN of the (A) normal group, (B) control group, (C) ATM100 group, (D) and ATM400 group. Coronal sections were 30 μm thick, and the scale bar represents 50 μm (100 × 100). Note: The results of immunohistochemistry were analyzed by performing separate one-way ANOVA of mean counts among the groups. Each value represents the mean ± SEM. *** *P *< 0.001 compared with normal and ^++^*P *< 0.01 compared with control.

The evaluation of the IL-1β–immunoreactive cells per section of the paraventricular area are shown in Figure 6A and B. The mean IL-1β–positive neuron count in the PVN area was 17.3 ± 0.8 in the normal group, 29.5 ± 2.2 in the control group, 18.3 ± 1.2 in the ATM100 group, and 17.3 ± 0.7 in the ATM400 group (F_3, 23 _= 21.3, *P* < 0.001). In the normal group, the mean IL-1β–positive neuron count was 170.5% that of the control group (*P* < 0.001). The LSD post hoc test revealed that the mean IL-1β–positive neuron count in the PVN was significantly lower in the ATM-treated groups compared with the control group (*P* < 0.01). Therefore, ATM administration was associated with suppression of IL-1β–positive neurons in the PVN.

## Discussion

We found that ATM administration was associated with decreased immobility time in the FST and decreased depression-like behavior in terms of social exploration and sucrose intake. ATM treatment was also associated with decreased serum CORT levels. Moreover, the brain expression of c-Fos-, TH-, and IL-1β–immunoreactive neurons was significantly reduced in the ATM-treated groups. These results align with the idea that ATM administration yielded antidepressant effects in this menopausal animal model.

The present study demonstrated that the OVX and repeatedly stressed female rats displayed significantly more anhedonic and learned helplessness behaviors and significantly less social exploration behavior and that these effects were diminished when ATM was administered to the rats*.* This study also showed that ovariectomy and repeated immobilization stress was associated with the CORT hypersecretion and imbalances of proinflammatory and anti-inflammatory cytokines in the serum. Stress-induced neuroinflammation appears to greatly depend upon stressors. For instance, stressors, such as the odor of a predator (Buchanan et al. 2008) and restraint stress (Hueston et al. 2011) induce inflammatory responses. Immobilization stress is among the main and potent sources of stress in humans, inducing strong immune and endocrine reactions (Savignac et al. 2011). Hypothalamic–pituitary–adrenal (HPA) axis hyperactivity is associated with increased proliferation of the proinflammatory cytokines IL-1 and IL-6 (Maes 1995; O'Connor et al. 2000; Lehtimaki et al. 2003). Interactions occur at multiple levels via the immune system, the autonomic nervous system, and the HPA axis (Petra et al. 2015). In line with the HPA axis theory of depression, cytokines also play roles in mediating the activity of the HPA axis (Chen et al. 2017). The present study also showed that IL-1β–immunopositive neurons were significantly increased in OVX and repeatedly stressed rats. The neuroanatomical substrates mediating stress responses include the supramammillary body (Choi et al. 2012), hippocampus (Park et al. 2010a; Park et al. 2010b) and hypothalamus (Park et al. 2009; Park et al. 2010a; Park et al. 2010b). In the central nervous system, IL-1β may stimulate the production of other cytokines (such as IL-6 or TNF-α) by astrocytes and microglia, promoting inflammatory processes in the brain (Maier and Watkins 1998). Both IL-6 and IL-1β are important in the pathophysiology of major depression (Yang et al. 2013). Zou et al. (2018) found that antidepressant drug intake was associated with changes in proinflammatory cytokine levels among patients with major depressive disorder. In the present study, we demonstrated that ATM administration was associated with suppressed IL-1β–immunoreactive neuron levels in the PVN in OVX and repeatedly stressed female rats*.* Other studies have reported that ATM regulates atopic dermatitis-like lesions (Yang et al. 2016), and its active flavonoids have been shown to suppress a TNF-α–related pathway in ethanol-induced liver injury and steatosis in mice (Lee et al. 2017). (Yu et al. 2010) used in vitro and in vivo experiments to demonstrate anti-inflammatory and anti-hepatitis effects associated with ATM administration.

ATM contains phenolic compounds and flavonoids among other active constituents. Isoamericanoic acid B extracted from ATM could be a promising phytoestrogen for use in the development of natural estrogen supplements. Additionally, the flavonoid has exhibited antidepressant effects in animals and in middle-aged and elderly women (Chang et al. 2016; German-Ponciano et al. 2018). Collectively, these findings suggest that ATM has significant anti-inflammatory effects via the regulation of proinflammatory cytokine expression. These results are consistent with previous studies and have demonstrated ATM’s anti-inflammatory effect in OVX and repeatedly stressed female rats.

ATM was found to exert anti-stress effects in the rats through the reduction of anhedonia and learned helplessness behaviors. It was also effective at regulating serum CORT levels as well as c-Fos and IL-1β expression in the brain. ATM may be a useful medicinal plant for controlling stress via peripheral and central stress modulators.
